# Suboptimal use of hormonal therapy among German men with localized high-risk prostate Cancer during 2005 to 2015: analysis of registry data

**DOI:** 10.1186/s12885-022-09677-z

**Published:** 2022-06-07

**Authors:** Semaw Ferede Abera, Ahmed Bedir, André Glowka, Dirk Vordermark, Daniel Medenwald

**Affiliations:** 1 Department of Radiation Oncology, Health Services Research Group, University Hospital Halle (Saale), Faculty of Medicine, Martin-Luther-University Halle-Wittenberg, Ernst-Grube-Str. 40, 06120 Halle (Saale), Germany; 2 Department of Radiation Oncology, University Hospital Halle (Saale), Faculty of Medicine, Martin-Luther-University Halle-Wittenberg, Ernst-Grube-Str. 40, 06120 Halle (Saale), Germany

**Keywords:** Hormonal therapy, High-risk, Prostate cancer, Treatment, Cancer registry, S3-guideline, Germany

## Abstract

**Background:**

This study assesses the use of hormonal therapy to treat high-risk localized prostate cancer (HRLPCa) cases diagnosed between 2005 and 2015.

**Methods:**

All N_0-X_M_0_ with ≥T_3a,_ or PCa cases with poorly differentiated feature (equivalent to Gleason score ≥ 8), diagnosed between 2005 and 2015 were extracted from German population-based cancer registries. Cases treated by surgery or chemotherapy were excluded. Description of hormonal therapy use by HRLPCa cases’ profile was presented. Relative risk (RR) was computed with a log-link function to identify factors associated with hormonal therapy use among radiotherapy-treated HRLPCa cases.

**Results:**

A total of 5361 HRLPCa cases were analyzed. Only 27.6% (95% confidence interval [CI]: 26.4–28.8%) of the HRLPCa cases received hormonal therapy in combination with radiotherapy. The use of combined hormonal therapy and radiotherapy varied from 19.8% in Saxony to 47.8% in Schleswig-Holstein.

Application of hormonal therapy was higher for the locally advanced cases compared to the poorly differentiated cases (relative risk [RR] = 1.28; 95%CI: 1.19, 1.37). Older patients showed a slightly increased use of hormonal therapy (RR for a 10-year age increase = 1.09; 95%CI: 1.02, 1.16). Compared to PCa cases from the most affluent residential areas, cases from the least affluent (RR = 0.71; 95%CI: 0.55, 0.92) and medium (RR = 0.75; 95%CI: 0.58, 0.96) areas had decreased use of hormonal therapy. The introduction of the German S3-guideline did not make a marked difference in the uptake of both hormonal therapy and radiotherapy (RR = 1.02; 95%CI: 0.95, 1.09).

**Conclusion:**

This study found a low use of hormonal therapy among HRLPCa patients treated without surgery. The introduction of the German S3-guideline for prostate cancer treatment does not seem to have impacted hormonal therapy use.

**Supplementary Information:**

The online version contains supplementary material available at 10.1186/s12885-022-09677-z.

## Background

Prostate cancer (PCa) is a malignant neoplasm of the prostate gland characterized by heterogeneous features and a variable natural history [1, 2]. PCa accounted for 22.7% (58,800) of the estimated 258,500 diagnosed cancer cases among German men in 2016. During the same year, with a predicted age-standardized incidence rate of 91.6 and a mortality rate of 19.5 per 100,000, PCa was an important cause of health problem in Germany [3]. Its treatment is costly, and adds a substantial economic burden to the German healthcare budget [4]. By 2030, PCa is projected to be the most frequent cancer in Germany, exceeding breast cancer [5]. An estimate of 15% PCa diagnoses are of high-risk disease, but what constitutes “high-risk” localized PCa (HRLPCa) varies in the literature [1, 6, 7]. In common with D’Amico et al., the “German S3-Guideline for Prostate Cancer” and the European Association of Urology (EAU)-guideline (see Additional file 11) define HRLPCa as having prostate specific antigens (PSA) at a level of > 20 ng/ml, a Gleason score (GS) ≥8, or clinical stage ≥ T2c [8, 9]. Discrepant with this definition, stage T2c cases without other high-risk features have shown better treatment outcomes than HRLPCa cases so classified in other ways; it has therefore been suggested that they be classified as intermediate-risk [10]. In line with these findings, the National Comprehensive Cancer Network (NCCN) guideline modified D’Amico’s definition of HRLPCa to include one or more of the following features: PSA > 20 ng/ml, biopsy GS ≥8, or clinical stage of > T2c [11]. In both the German S3-guideline and EAU, the term “locally advanced” PCa has been used to refer to a subgroup of HRLPCa with T_3-4_N_0_M_0_ clinical features [9, 12]. Since the data underlying this study lack records of PSA values, the focus of our investigation is only on HRLPCa cases with GS ≥8 or clinical stage ratings higher than T2c.

HRLPCa has a high chance of developing distant metastases or of not responding to treatment, either of which increases the risk of PCa-specific mortality [1, 8]. In the context of multimodal therapeutic intervention, a combination of long-term hormonal therapy (HT) and external-beam radiation therapy (EBRT) is the standard treatment for men with HRLPCa disease, although radical prostatectomy is also an optional major mode of treatment [1, 12–21]. Long-term androgen HT synergistically potentiates EBRT, and their combination is superior to radiotherapy (RT) or HT alone [13, 14, 17]. According to the evidence- and consensus-based interdisciplinary German S3-Guideline and the EAU-guideline, HRLPCa cases should be treated by a combination of long-term HT and RT, or surgery [9]. Similarly, the European guideline recommends external irradiation in combination with long-term HT as the standard treatment modality for high-risk localized and locally advanced PCa patients [12]. Additional file 11 summarizes the definition of HRLPCa and the detailed treatment recommendations of both the German S3-guideline (2009 to 2021) and EAU guideline (2005 to 2020). Despite the well-substantiated evidence and guideline recommendations that HT should be the mainstay adjuvant treatment for HRLPCa-treated with RT [9, 11, 12, 14, 21], under-treatment of high-risk PCa cases is a concern [6, 22, 23]. In addition to having direct negative consequences for patients, clinical practices which diverge from guidelines have been reported to incur unnecessary expenses [24].

The objective of our study is to assess the use of HT to treat patients with HRLPCa diagnosed between 2005 and 2015, using data from the German population-based cancer registries. In addition to presenting HT use in relation to the clinical characteristics of incident cases, we examine the predictors of HT use among the RT-treated subgroup. The effects of area-based socio-economic position and the introduction of the German S3-guideline for prostate cancer on the use of HT are also assessed.

## Methods

### Data source description and study population

Population-based cancer registries are crucial sources of information for cancer epidemiology and health services research. Following the enactment of the Federal Cancer Register Data Act (Bundeskrebsregisterdatengesetz, BKRG) in 2009, the German Center for Cancer Registry Data (Zentrum für Krebsregisterdaten, ZfKD) was set up at the Robert Koch-Institute [25]. By law, all federal states were obliged to collect cancer registry data.

In brief, the state cancer registries collect data on key case demographics such as gender, month and year of birth, and area of residence; data about the tumor at time of diagnosis including date of diagnosis, tumor topography and morphology, and tumor grading and stage; and data on delivered treatments, death events, and cause of death for deceased cases. Comprehensive data are also collected from clinical cancer registries (CCRs). The population-based state cancer registries are currently responsible in most cases for both the population-based and clinical cancer registries [26], although only the population-based, and not the CCRs, data are transferred to the ZfKD. On receiving data from the state population-based cancer registries, the ZfKD checks the data quality, pools the data, and produces nationwide and regional reports. The ZfKD also provides anonymized data to external users upon request [3]. Details on techniques of data quality assessment, and procedures for data request can be found on the web page [27].

This study used pooled nationwide HRLPCa data, representing all diagnoses from 2005 to 2015. Similar to Hager et al. [28], we included only state cancer registries which had diagnosis and basic treatment data for at least 70% of their registered cases since 2005. Only seven federal states (Schleswig-Holstein, Berlin, Brandenburg, Mecklenburg-Vorpommern, Saxony, Saxony-Anhalt, and Thuringia) met these inclusion criteria. Data from Berlin and Saxony-Anhalt were not included in the main analysis since both states had relatively low numbers of HRLPCa cases. All cases treated by surgery and/or chemotherapy, or for which diagnosis was only by death certificate or autopsy, were also excluded. Specific conditions of high-risk PCa cases, like cases with limited life expectancy (< 10 years), could have led to their being under- or over-treated, which could affect our analysis [29]. Because of this likely problem, only those cases with sufficient life expecancy (> 10 years) were included [30]. Estimation of life expectancy (stratified by age and calendar period) for the German male population was obtained from the Human Mortality Database [30]. We assumed that the HRLPCa cases would have comparable life expectancy with the age group- and calendar period- matched German population, had they received the recommended treatment standard. Because of the limited life expectancy, all HRLPCa cases in individuals over 79 years old were excluded from this study. These excluded cases are also likely to suffer from comorbidities, such as cardiovascular diseases, a clinical scenario that may prevent prescription of HT in clinical practice. The exclusion of these cases was, therefore, methodologically relevant.

### Measurements and statistical analysis

All N_0-X_M_0_ PCa (ICD-10 C61) cases with ≥T_3a_ cases or histopathological tumor grades 3, 4, and 7 according to the coding system of the registries, which were equivalent to a Gleason’s score of eight or higher [31], were included. The term ‘locally advanced PCa’ refers to PCa cases that harbored ≥T3a feature [9, 12]. Cases with both poor differentiation and locally advanced characteristics were considered locally advanced. Those cases, which were diagnosed before and after September 2009, were considered as “diagnosed before the era of the German S3-guideline for prostate cancer treatment”, and “diagnosed during the era of the German S3-guideline” respectively [32].

In this study, non-treatment was constituted by receiving neither RT nor HT in the included HRLPCa cases. The German Indicator for socio-economic deprivation (GISD) was used to measure regional socio-economic deprivation [33]. GISD data were collected at German municipal, administrative district, and regional levels. We extracted data from the recommended, 2018-updated version, which covers the period 1998 and 2014 [34]. This GISD version did not include data for 2015. Therefore, district-level GISD data, covering 2005 to 2014, were linked with the prostate cancer registry data to model factors of HT use*.* Comprehensive methodological approaches are detailed elsewhere [33]. Calendar year-specific quintile classifications were created from the total GISD score. The degree of socio-economic deprivation increases with quantile increase; quantile 1 represents the least deprived group, whereas quantile 5 represents the least affluent group. In our model, the lowest quintile was classified as “most affluent”, the middle three quintiles (quintiles 2, 3 and 4) as “medium”, and the highest quintile as the “least affluent” [33].

The dependent variable was use of hormonal therapy among RT-treated HRLPCa cases. Age at time of diagnosis, state, GISD, stage, tumor grade, and era of diagnosis (before or during S3-German guideline era) were the independent variables. Multivariable log-binomial model was used to identify factors associated with HT use among 2349 RT-treated HRLPCa cases from the five federal states (Schleswig-Holstein, Brandenburg, Mecklenburg-Vorpommern, Saxony, and Thuringia) whose data met our criteria, see Additional file 1. In order to assess the robustness of the estimated relative risks, we performed a sensitivity analysis on the data that additionally included Berlin and Saxony-Anhalt (Additional file 3). Predictors of missing treatment and tumor grade information were assessed using multivariable binary logistic regression (Additional file 6 and Additional file 8). The statistical analyses were carried out using Stata 15.1 (Stata Corp, College Station, TX, USA). Maps of regional HT use were plotted in R.

## Results

### Description of the incident case population by treatment

A total of 5361 HRLPCa cases were included from five federal states, of which 3546 (66.1%) and 1815 (33.9%) were poorly differentiated and locally advanced PCa cases, respectively. The median age was 73 years (range, 42 to 79; interquartile range, 69 to 76). Majority of the cases, 3306 (61.7%), were from Saxony (35.7%), and Brandenburg (26%), together. GISD information was available for 4933 cases (92%), and just over half, 2566 (52%), of them were living in the least affluent residential areas (Table 1).

**Table 1:** Prostate cancer treatment distribution for localized poorly differentiated and locally advanced PCa cases diagnosed between 2005 and 2015 by treatment status (*n =* 5, 361)

Variables	Levels	Total number of cases	Received RT only (n, %)	Received HT only (n, %)	Received both treatments (n, %)	Received none of the treatments (n, %)
Age at diagnosis, median (IQR)	73 (69–76)		73 (68–75)	74 (70–77)	73 (69–76)	73 (68–76)
Stage	Poorly differentiated	3, 546	804 (22.7)	1042 (29.4)	974 (27.4)	726 (20.5)
	Locally advanced	1, 815	238 (13.1)	741 (40.8)	507 (27.9)	329 (18.1)
Tumor grade ^a^	Low	461	59 (12.8)	196 (42.5)	129 (28.0)	77 (16.7)
	High	4, 683	950 (20.3)	1500 (32.0)	1315 (28.1)	918 (19.6)
German index of socio-economic deprivation (GISD)^b^	Wealthiest	47	8 (17.0)	13 (27.7)	20 (42.5)	6 (12.8)
	Wealthy	275	55 (20.0)	98 (35.6)	82 (29.8)	40 (14.5)
	Medium	306	114 (37.2)	52 (17.0)	123 (40.2)	17 (5.6)
	Poor	1, 739	277 (15.9)	662 (38.1)	455 (26.2)	345 (19.8)
	Poorest	2, 566	502 (19.6)	662 (32.9)	713 (27.8)	513 (20.0)
S3-Guideline era	Pre-guideline era	1, 834	320 (17.4)	838 (32.6)	532 (29.0)	284 (15.5)
	Guideline era	3, 527	722 (20.5)	1085 (30.7)	949 (26.9)	771 (21.8)
German federal states	Schleswig-Holstein	701	261 (37.2)	82 (11.7)	335 (47.8)	23 (3.3)
	Brandenburg	1, 393	239 (17.2)	536 (38.5)	412 (29.6)	206 (14.8)
	Mecklenburg-Vorpommern	726	137 (18.9)	192 (26.4)	210 (28.9)	187 (25.8)
	Saxony	1, 913	173 (9.0)	860 (45.0)	378 (19.8)	502 (26.2)
	Thuringia	628	232 (36.9)	113 (18.0)	146 (23.3)	137 (21.8)
Total		5, 361	1042 (19.4)	1783 (33.3)	1481 (27.6)	1055 (19.7)

*IQR* Interquartile range, % Row percentage, ^a^ grading information was missed for about 4.05% (217) of the 5, 361 cases, ^b^ GISD information was available for 4, 933 observations, *RT* Radiotherapy, *HT* Hormonal therapy

The mean proportion of hormonal therapy (HT) use, regardless of RT treatment status, was 57.8%, varying from 41.2% in Thuringia to 68.1% Brandenburg. As shown in Table 1, only 27.6% (95% confidence interval [CI]: 26.4–28.8%) of the HRLPCa cases received HT in combination with radiotherapy (RT). This proportion varied among states, ranging from about one-fifth, 378 (19.8%), in Saxony to about half, 335 (47.8%), in Schleswig-Holstein. Compared to other states, Schleswig-Holstein and Brandenburg had the highest proportions of cases treated using combined hormonal and radiation treatment, 47.8 and 29.6% respectively (*P* < 0.001).

Nearly one-fifth, 19.7% (95% CI: 18.6–20.8%), of HRLPCa cases did not receive either RT or HT. The proportion of cases that received neither treatment was higher during the guideline era (post 2009) than the pre-guideline era (15.5% vs. 21.9%, *P <* 0.001). Non-treatment was slightly higher in the poorly differentiated cases compared with locally advanced cases (20.5% vs. 18.1%, *P* < 0.041). It was also higher for cases from Saxony (26.2%) and Mecklenburg-Vorpommern (25.8%), whereas Schleswig-Holstein (3.3%) documented the lowest proportion. The proportion of non-treatment was 7.2% lower for the most affluent group compared to the least affluent group with (12.8% vs. 20%, *P* = 0.034) (data not shown).

### Treatment patterns

**Fig. 1:**
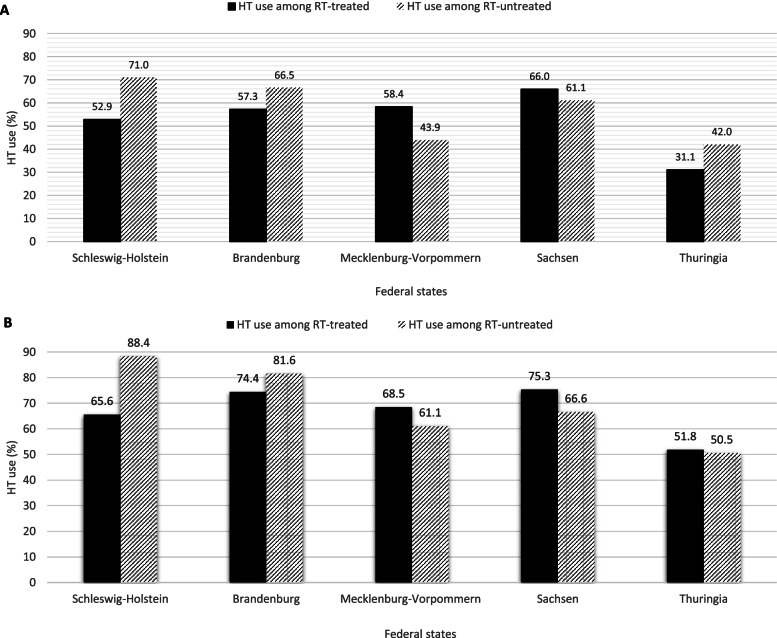
Proportion of HT use by German states among RT-treated and RT-untreated cases. (**A**) Poorly differentiated prostate PCa cases (**B**) Locally advanced PCa cases

Figure 1 shows the proportion of HT used for both RT-treated and -untreated HRLPCa cases in five federal states of Germany from 2005 to 2015. The use of HT among RT-treated, poorly differentiated group ranges from 31.1% in Thuringia to 66% in Saxony. Poorly differentiated PCa cases were more likely to receive HT alone than a combination of HT and RT, except those poorly differentiated cases from Mecklenburg-Vorpommern and Saxony (Fig. 1A). A similar pattern was also observed for the locally advanced cases, but not in Thuringia (Fig. 1B). In all the five states, the locally advanced group, compared to the poorly differentiated group, more frequently received HT. On average, the percentage use of HT among the RT-treated, locally advanced cases was 14% higher than the poorly differentiated cases of the same treatment group. Similarly, the mean percentage was 12.7% higher for the RT-untreated, locally advanced cases than the same treatment group in the poorly differentiated cases. The highest proportion of use of combined HT and RT to treat locally advanced cases was observed in Saxony and Brandenburg, while the lowest was in Thuringia (Fig. 1B).

**Fig. 2:**
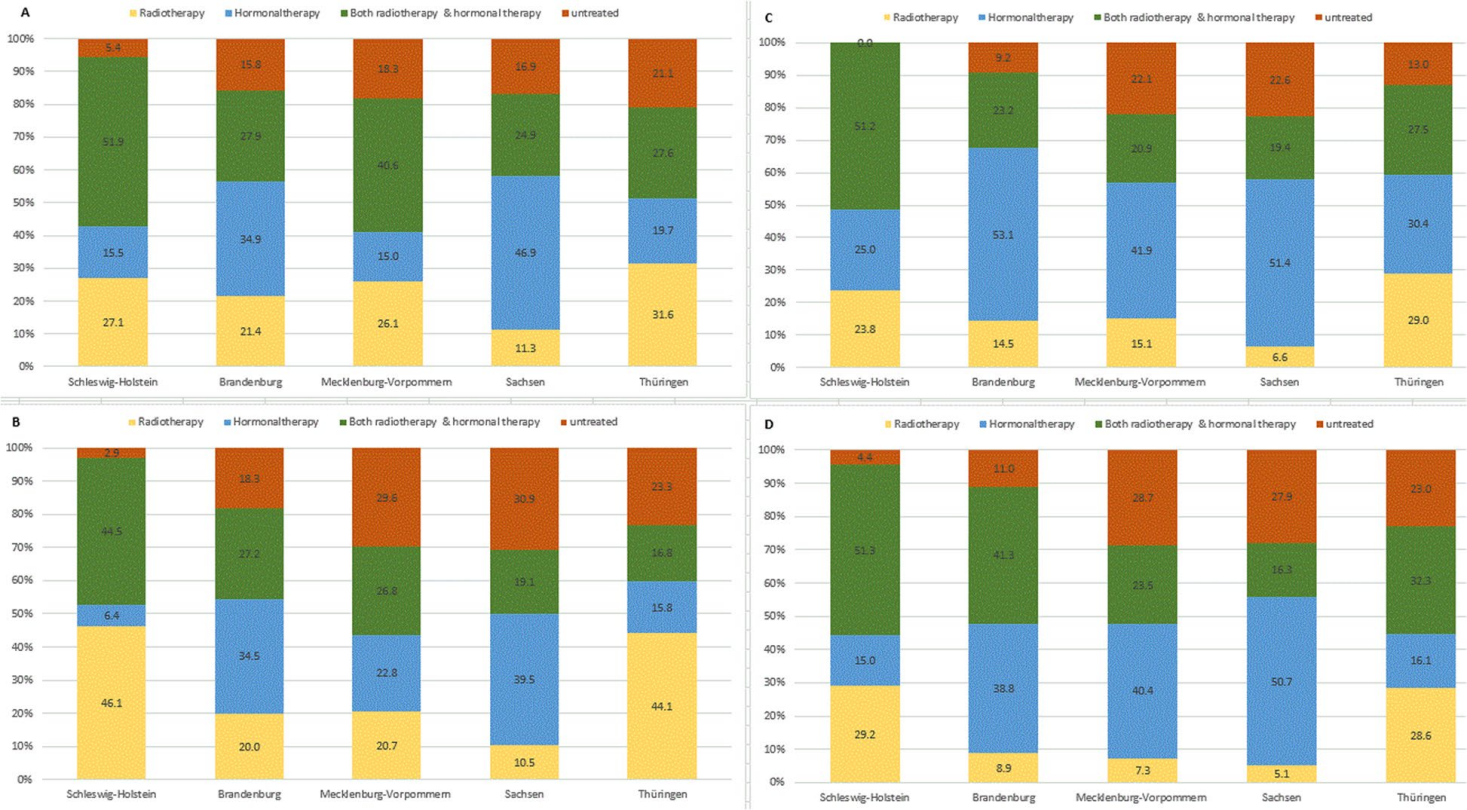
Treatment trends of high-risk PCa cases by state and German S3-Guideline era, 2005-2015. (**A**) Treatment trend of poorly differentiated cases before guideline era. **B** Treatment trend of poorly differentiated cases during guideline era. **C** Treatment trend of locally advanced cases before guideline era. **D** Treatment trend of locally advanced cases during guideline era

Figure 2 presents the proportions of HT use in HRLPCa cases of the five federal states, stratified by PCa German S3-Guideline era and RT treatment status. The share of cases for which HT was used in combination with RT was highest in Schleswig-Holstein in both PCa groups, regardless of guideline implementation era. Use of both treatments in combination was consistently lowest in Saxony, with the single exception of the poorly differentiated group during the guideline era, where Saxony (19.1%) had the second lowest rank next to Thuringia (16.8%). For the poorly differentiated group, the mean percentage use of combined HT and RT before and during the guideline era were 34.6 and 26.9%, respectively. However, there was a 4.5% increase (from 28.4 to 32.9%) in the mean percentage use of combined HT and RT during the guideline era by the locally advanced group (Fig. 2). The proportion of untreated HRLPCa cases was higher during the guideline era, compared to the pre-guideline era. Schleswig-Holstein had the lowest proportion of untreated cases (Fig. 2).

**Fig. 3:**
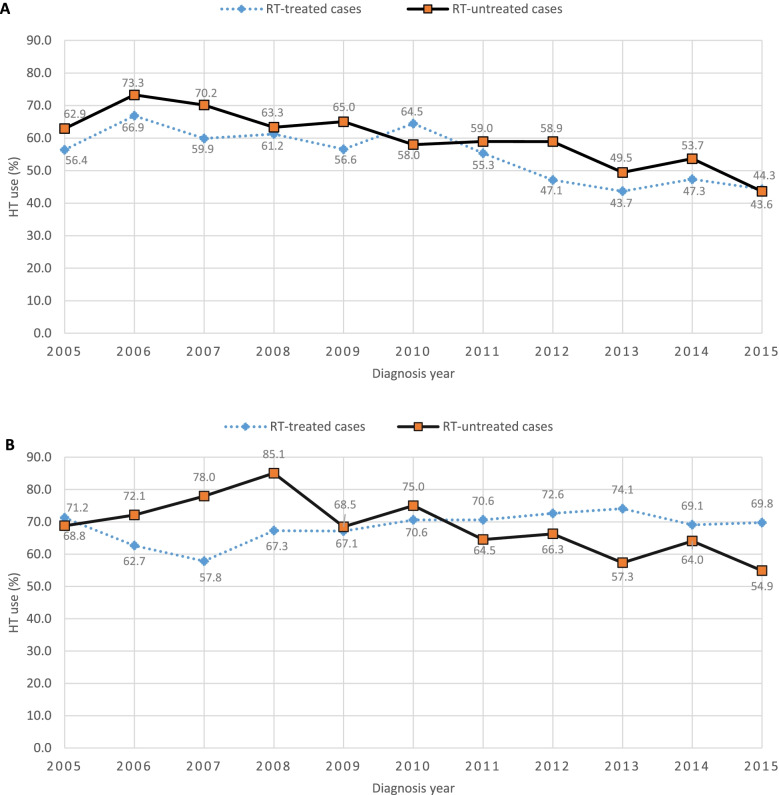
Trend of HT use among RT-treated and -untreated cases by year of diagnosis. (**A**) Poorly differentiated prostate PCa cases (**B**) Locally advanced PCa cases

The use of HT among RT-treated, poorly differentiated cases was lower than the use of HT in cases which were not treated by RT across all the years, with the two annual exceptions of 2010 and 2015. Additionally, the proportion of HT use for both the RT-treated and -untreated poorly differentiated cases appears to decline starting from 2011 (Fig. 3A). In contrast to the poorly differentiated group, in locally advanced cases the use of combined HT and RT has been relatively higher since 2010, compared to the RT-untreated cases (Fig. 3B).

**Fig. 4:**
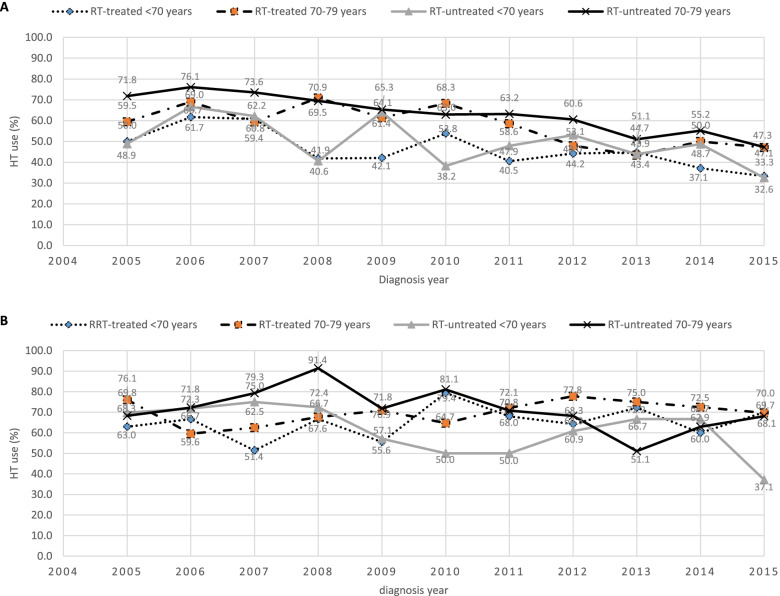
Trend of HT use by age group and year of diagnosis among RT-treated and RT-untreated cases. (**A**) Poorly differentiated PCa cases (**B**) Locally advanced PCa cases

Use of HT was higher in older cases (age at diagnosis) in both poorly differentiated and locally advanced cases, as well as among RT-untreated cases within the same age group (Fig. 4A-B). The locally advanced cases had higher use of HT compared to the poorly differentiated cases (Fig. 4A-B).

**Fig. 5:**
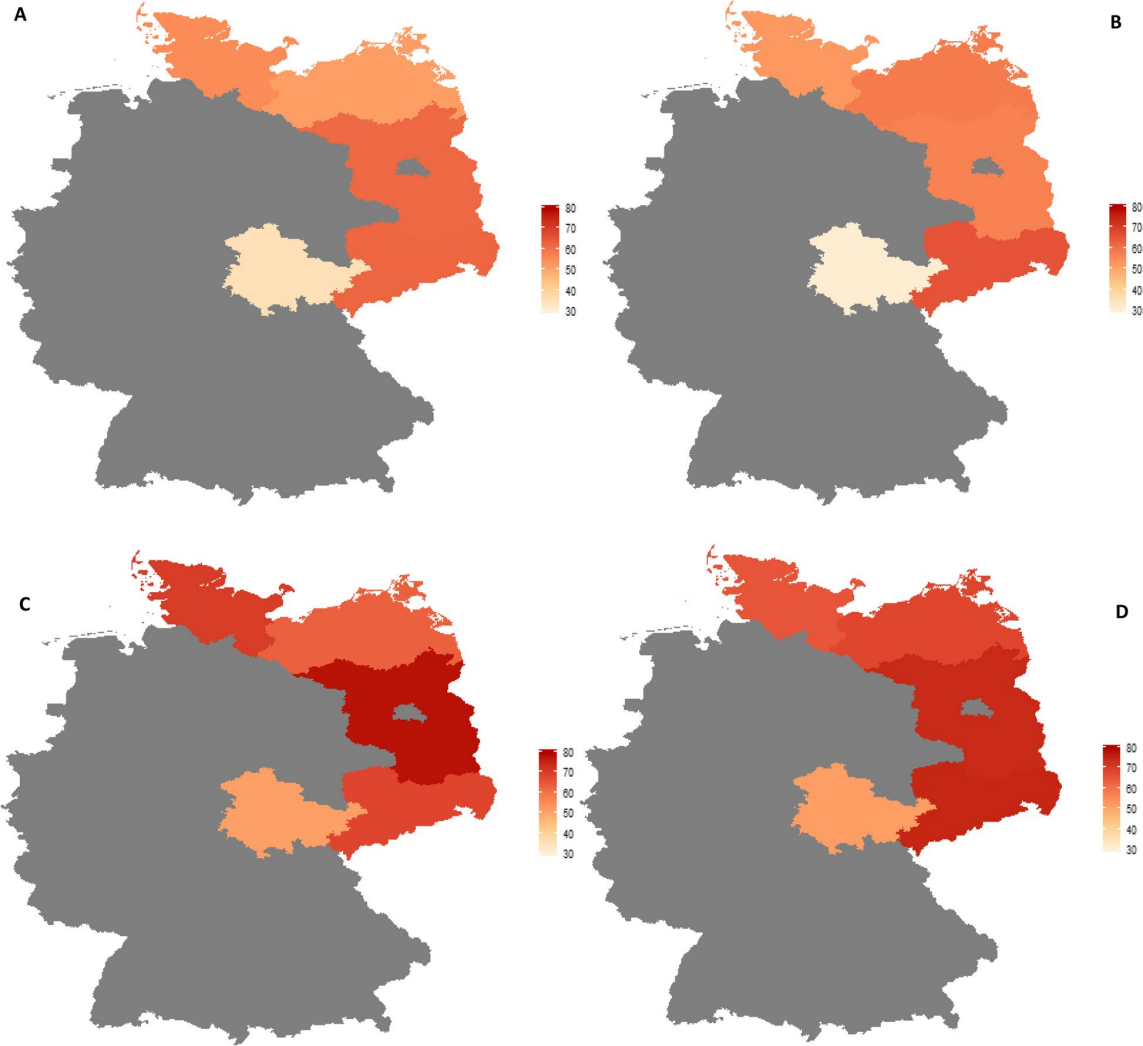
Proportion of HT use among HRLPCa cases by RT treatment status in five German states. (**A**) All poorly differentiated cases among RT-treated and -untreated cases (**B**) Poorly differentiated cases that received RT (**C**) All locally advanced among RT-treated and -untreated cases (**D**) Locally advanced cases that received RT

The proportions of HT use in the five federal states, stratified by RT treatment status, are summarized in Fig. 5. In general, use of HT was higher for treatment of locally advanced cases. For poorly differentiated cases, the use of HT in combination with RT was highest in Saxony (Fig. 5B), and Saxony and Brandenburg also showed highest proportions of use of HT in combination with RT for the locally advanced cases (Fig. 5D). Data from the seven federal states also showed similar results (Additional file 2).

### Predictors of hormonal therapy use among HRLPCa cases treated by radiotherapy

Table 2 presents the univariable and multivariable regression results. The multivariable log-binomial model identified that locally advanced PCa cases compared to the poorly differentiated cases (RR = 1.28: 95%CI: 1.19, 1.37) were associated with increased use of HT. For every 10-year increase in the patients’ age, there was a slight increase in the use of HT (RR = 1.09; 95%CI: 1.02, 1.16).

**Table 2:** Clinical and socio-demographic factors associated with HT use among poorly differentiated and locally advanced PCa cases which received RT between 2005 and 2014 (*n =* 2, 349)

Variables	Received HT		Use of HT	
	No (n, %)	Yes (n, %)	Crude Risk Ratio (95% CI)	Adjusted Risk Ratio (95% CI)
**Age (10 year increase)**			1.08 (1.01, 1.15)	1.09 (1.02, 1.16)
**Tumor grade**^**a**^				
Low grade	56 (31.6)	121 (68.4)	1.00	1.00
High grade	867 (41.3)	1, 235 (58.7)	0.86 (0.77, 0.96)	1.07 (0.96, 1.19)
**Stage**				
Poorly differentiated	731 (44.4)	916 (55.6)	1.00	1.00
Locally advanced	225 (32.1)	477 (67.9)	1.22 (1.14, 1.31)	1.28 (1.19, 1.37)
**German Index of Socioeconomic-deprivation**^**b**^				
Most affluent	8 (28.6)	20 (71.4)	1.00	1.00
Medium	446 (40.3)	660 (59.7)	0.84 (0.66, 1.06)	0.75 (0.58, 0.96)
Least affluent	502 (41.3)	713 (58.7)	0.82 (0.65, 1.04)	0.71 (0.55, 0.92)
**Era**				
Pre-guideline era	375 (37.7)	620 (62.3)	1.00	1.00
Guideline era	581 (42.9)	773 (57.1)	1.09 (1.02, 1.17)	1.02 (0.95, 1.09)
**Federal States**				
Schleswig-Holstein	238 (43.1)	314 (56.9)	1.00	1.00
Brandenburg	227 (36.7)	392 (63.3)	1.11 (1.01, 1.22)	1.10 (1.00, 1.22)
Mecklenburg-Vorpommern	131 (40.7)	191 (59.3)	1.04 (0.93, 1.17)	1.09 (0.96, 1.23)
Saxony	163 (31.2)	360 (68.8)	1.21 (1.10, 1.33)	1.21 (1.10, 1.33)
Thuringia	197 (59.2)	136 (40.8)	0.72 (0.62, 0.83)	0.72 (0.62, 0.83)

^a^ grading information was missed for about 2.98% (70) of the 2349 cases, ^b^ GISD information available only until 2014

Based on the German Index for Socioeconomic-Deprivation classification, HRLPCa cases from medium (RR = 0.75; 95%CI: 0.58, 0.96), and least affluent (RR = 0.71; 95%CI: 0.55, 0.92) residential areas had decreased HT use compared to those from the most affluent areas (Table 2).

The model from the sensitivity analysis, based on the seven German states, generally showed similar results demonstrating the robustness of the main estimates (Additional file 3). Compared to Schleswig-Holstein, the use of HT was lower in Berlin (RR = 0.68; 95%CI: 0.54, 0.84) and Thuringia (RR = 0.72; 95%CI: 0.62, 0.83).

Additional file 4 shows that higher age was inversely associated with non-treatment in both the poorly differentiated (RR = 0.79; 95%CI: 0.71, 0.88), and locally advanced cases (RR = 0.84; 95%CI: 0.71, 0.99); patients were less likely to undergo treatment the older they were, regardless of how sick they were. Poorly differentiated cases diagnosed during the guideline-era were 1.42 times more likely (RR = 1.42; 95%CI: 1.22, 1.65) to risk of non-treatment, compared with cases diagnosed before the guideline-era, but no evidence of a strong association was observed for the locally advanced cases (Additional file 4).

### Sensitivity analysis

A sensitivity analysis based on 778 PCa cases, excluding cases with tumor code grade 3 in the registry regardless of their tumor stage, showed that the proportion of use of HT in combination with RT was 24.3% (95%CI: 21.4–27.4%). The relative risk estimates from the sensitivity analysis performed on 2648 RT-treated HRLPCa cases were similar to the estimates from the main model (Additional file 3). This reflected the stability of the estimated risk-ratio from the main model. Moreover, increasing age, year of diagnosis (2011–2015 vs. 2005–2010), and missing RT data were predictors of missing hormonal treatment data (Additional file 6). Generally, increasing age was a strong predictor for missing TNM-stage and histopathological tumor grade (Additional files 8 and 9). While year of diagnosis (2011–2015 vs. 2005–2010) was associated with missing tumor grade data, it was inversely associated with missing TNM-stage data (Additional files 8 and 9). Unfortunately, the state-specific odds ratio estimates for missing PCa stage data were not precise due to few numbers of missing observations in each state (Additional file 8). Additional file 5 summarizes the proportions of missing treatment data, stratified by German federal states, for all the non-metastatic PCa cases.

## Discussion

In this study, we assessed the clinical practice of prescribing HT for localized high-risk PCa cases in the context of prostate cancer treatment in Germany. We have found that only 27.6% (95%CI: 26.4–28.8%) of the 5361 HRLPCa cases not treated by surgery or chemotherapy received HT in combination with RT. Older age and non-affluent residential area were associated with increased risk of non-use of HT among HRLPCa cases which also received RT. However, locally advanced tumors were associated with increased use of HT compared to the poorly differentiated tumors. Another key finding was that nearly one in five cases were untreated.

Evidence-based guidelines, if successfully implemented and regularly revised with up-to-date evidence, may play a crucial role in improving clinical practice and treatment outcomes [35, 36]. Following objectively defined procedures, Germany developed its evidence and consensus based guideline, and published its first version in 2009 [9, 32]. This guideline recommends that HRLPCa cases be treated either by radical prostatectomy and adjuvant RT, or long-term HT in combination with EBRT [9, 18, 19]. This study assessed the use of HT in relation to the second treatment option. Multiple randomized control trials have demonstrated that long-term HT in combination with RT for treating high-risk localized and locally advanced PCa patients showed superior oncological outcomes such as improved overall survival, reduced diseases progression and biochemical failure [13, 17–20, 37, 38]. In our study, however, only approximately one quarter of the surgically untreated HRLPCa cases received guideline-recommended treatment. This finding was far below those reported in studies in the U.S.A. [22, 39–41] and in the Netherlands [42]. Contrary to our study, data from German Prostate Cancer Centers found high guideline-adherence (85.4% in 2017) in terms of delivering RT with HT for locally advanced (T_3-4_N_0_M_0_) PCa cases [43]. Similarly, a study based on 70,683 patients treated in certified prostate cancer treatment centers between 2010 and 2013, found high fulfilment of quality requirement for more than 80% of the certified treatment centers [44]. Our population-based cancer registry data suggest that the target of achieving more than 90% for the use of RT with HT [43], an important quality indicator of PCa treatment, appears to be off track. In particular, the fact that HT use did not increase after the introduction of the German S3-guideline for Prostate Cancer treatment was highly unanticipated. Before the German S3-guideline became available, the EAU-guideline also recommended that HRLPCa cases receive a combination of HT and RT, although the duration of HT for the localized high-risk cases was mostly restricted to 6 months (Additional file 11). Regrettably, desired clinical outcomes are not achieved simply by publishing clinical guidelines [24]. A key finding of the current study, suboptimal HT use, may suggest low adherence to the guideline. Whether the observed HT underutilization was related to poor guideline adherence or to treatment underreporting remains unanswered by the data underlying our results, and further study may need to be undertaken. Prior studies indicate that adherence to guideline recommendations has been a concern, and several barriers may affect guideline use in clinical practice [24, 35]. It has been shown that discordance from PCa guideline may cause unfavorable effects at the patient- and health system-levels [24].

Comorbidity, patient refusal, advanced age and, in some cases, failure to initiate by physicians were mentioned as causes of non-prescription of HT among eligible PCa cases treated in certified prostate cancer treatment centers in Germany [45]. Since patients with PCa are a generally older population, the likelihood of comorbidity could also be higher in our study population. If this holds true, we could suppose that occurrence of comorbidities might have contributed for the decreased uptake of HT in the HRLPCa cases. While some studies on the association of comorbidity and HT use showed conflicting results [23, 42, 46], absence of comorbidity data in this study makes the interpretation of our results difficult. Wang et al. found a decrease in the duration of HT was certainly related to comorbidities, but it was mentioned that the degree of HT underutilization was not fully explained by comorbidities alone [40]. On the other hand, regional differences in the translation of evidence into clinical practice, rather than patient-related factors such as comorbidity, were deemed to be a possible cause of regional variation in the U.S. [46]. For instance, prescription of HT was more affected by the practices of individual urologists than by tumor- or patient-related characteristics [47]. HT prescription was also shown to vary by institutional factors, like differences in institutional policy, or whether the treating institutions are public or private [42, 48]. Similar to our study, decreasing patterns of HT use were observed in the U.S. [22, 23], but a reverse pattern was found in Australia [48]. Another important factor that could influence uptake of HT is the modality of radiation used for treatment. High-risk PCa cases which received brachytherapy were observed to experience lower odds of receiving HT [39]. However, the overall use of brachytherapy in Germany has been less than 2% [49], and thus brachytherapy is unlikely to influence the observed underutilization of HT.

In this study, the threat of underutilization of HT among HRLPCa is the most important clinically relevant finding, and this did not show improvement after the introduction of the German S3-guideline. It is also important to point out that the German S3-guideline suggests that localized, intermediate-risk PCa cases should be treated with a combination of EBRT and short-term HT [9, 45]. However, further classification of the intermediate-risk PCa cases into favorable and unfavorable groups has clinical importance [50]. A combination of RT and short-term HT is the optimal treatment for unfavorable intermediate-risk PCa cases, but not necessarily for favorable cases. The German S3-guideline did not introduce this classification scheme during the treatment period now studied (2005 to 2015) and adoption of this scheme could be beneficial for PCa patients, at least by avoiding HT overtreatment among the favorable intermediate-risk PCa cases [50].

There are additional limitations that should be considered when using the results of this study. The data had high proportions of missing diagnostic and treatment information (Additional files 1, 5 and 7). We therefore tried to assess the potential impact of these missing data on our main estimates. Additional file 3 shows that the main relative risk estimates presented in Table 2 are robust. It is important to note that missing hormonal treatment data depended on age, missing RT data, and year of diagnosis. On the other hand, the cancer registries in the former East Germany did not have missing values. This is because only delivered treatments were actively recorded by the respective cancer registries, and hence “no therapy” became the default record value. It is possible that HRLPCa cases might actually have received RT or HT, but treatment data were not submitted to the registries, and hence their treatment status was recorded as “no therapy”. On the other hand, possible side effects of HT might have been a barrier to its uptake [20]. In this study, all federal states with more than 30% missing diagnostic and treatment data were excluded. The five states we included for the main analysis had higher mean socio-economic deprivation compared to the excluded states (Additional file 10). That being the case, selection bias could be a potential limitation of this study, and our results may not reflect the situation of HT use in the excluded German states.

## Conclusions

In conclusion, this study assessed the status of HT use in surgically-untreated HRLPCa cases using population-based cancer registry data in selected states of Germany. Despite its limitations, our investigation showed a high likelihood of underutilization of HT in the non-metastatic HRLPCa cases between 2005 and 2015. The introduction of the German S3 treatment guideline for prostate cancer did not markedly affect HT use. This may reflect evidence of sub-optimal guideline adherence.

## Supplementary Information

**Additional file 1.** Inclusion and exclusion criteria

**Additional file 2.** Proportion of HT use among poorly differentiated and locally advanced PCa cases by RT treatment status in seven federal states of Germany, 2005–2015. (A) All poorly differentiated cases among RT- treated and -untreated cases (B) Poorly differentiated cases that received RT (C) All locally advanced among RT- treated and -untreated cases (D) Locally advanced cases that received RT.

**Additional file 3 **Factors associated with HT use among poorly differentiated and locally advanced PCa cases in seven states which received RT between 2005 and 2014 (*n =* 2, 648).

**Additional file 4 **Factors associated with non-treatment among poorly differentiated (*n =* 3, 243) and locally advanced (*n =* 1, 690) PCa cases diagnosed between 2005 and 2014.

**Additional file 5 **Proportions of missing treatment data among non-metastatic PCa cases stratified by German federal states, 2005–2015 (*n =* 263,839)

**Additional file 6 **Multivariable binary logistic regression analysis showing predictors of missing hormonal treatment data in Schleswig-Holstein (*n =* 767)

**Additional file 7 **Proportions of missing TNM stage data in non-metastatic PCa cases stratified by German federal states, 2005–2015 (*n =* 263, 839)

**Additional file 8 **Multivariable binary logistic regression analyses showing predictors of missing stage data in five states, 2005–2015 (*n =* 74,098)

**Additional file 9 **Multivariable binary logistic regression analyses showing predictors of missing histopathological tumor grade data in five states, 2005–2015 (*n =* 74,098)

**Additional file 10.** Mean GISD score of 16 German federal states based on 263,774 PCa cases diagnosed during 2005–2014 (1 = Schleswig-Holstein, 2 = Hamburg, 3 = Lower Saxony, 4 = Bremen, 5 = North Rhine-Westphalia, 6 = Hessen, 7 = Rhineland-Palatinate, 8 = Baden-Württemberg, 9 = Bavaria, 10 = Saarland, 11 = Berlin, 12 = Brandenburg, 13 = Mecklenburg-Vorpommern, 14 = Saxony, 15 = Saxony-Anhalt, and 16 = Thuringia)

**Additional file 11.** Treatment recommendations of German S3-Guideline and EAU guidelines for localized high-risk prostate cancer patients, 2005 to 2021

## Data Availability

The data that support the findings of this study, epidemiological data from selected German population-based cancer registries, were obtained from Robert Koch Institute (RKI). As the authors do not own the dataset, they cannot make it publicly available.

## Abbreviations

BKRG: Bundeskrebsregisterdatengesetz (Federal Cancer Register Data Act)
CCRs: Clinical cancer registries
EAU: European Association of Urology
EBRT: External-beam radiation therapy
GISD: German indicator for socio-economic deprivation
GS: Gleason score
HRLPCa: High-risk localized prostate cancer
HT: Hormonal therapy
ICD-10: International classification of diseases 10th version
IQR: Interquartile range
NCCN: National Comprehensive Cancer Network
PCa: Prostate cancer
PSA: prostate specific antigen
RT: Radiotherapy
TNM-stage: Tumor, node, metastasis stage
U.S.: United states
ZfKD: Zentrum für Krebsregisterdaten (Center for Cancer Registry Data)
