# Supplementary figures and images for: Suboptimal use of hormonal therapy among German men with localized high-risk prostate Cancer during 2005 to 2015: analysis of registry data
Source: BMC Cancer. 2022 Jun 7;22:624. doi: 10.1186/s12885-022-09677-z (PMC9171996; doi:10.1186/s12885-022-09677-z)

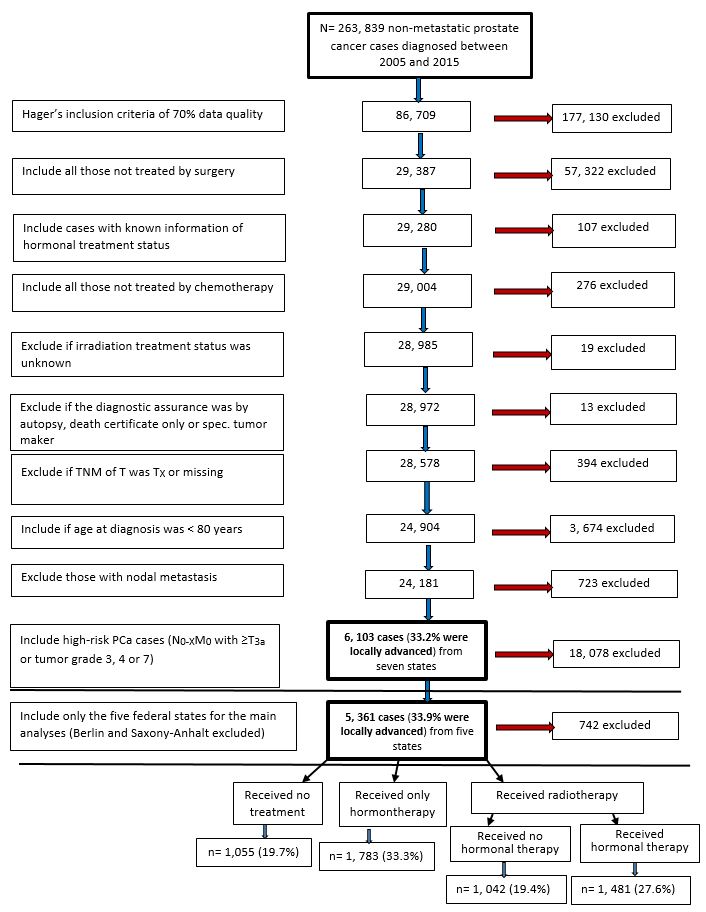

Supplement: Supplementary file 1 — Additional file 1. Inclusion and exclusion criteria [file 12885_2022_9677_MOESM1_ESM.png]

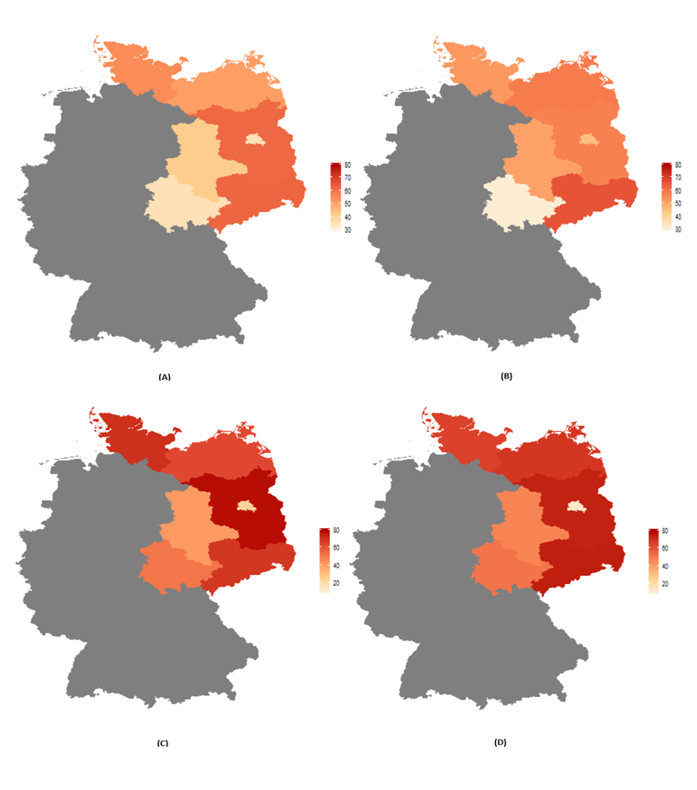

Supplement: Supplementary file 2 — Additional file 2. Proportion of HT use among poorly differentiated and locally advanced PCa cases by RT treatment status in seven federal states of Germany, 2005–2015. (A) All poorly differentiated cases among RT- treated and -untreated cases (B) Poorly differentiated cases that received RT (C) All locally advanced among RT- treated and -untreated cases (D) Locally advanced cases that received RT. [file 12885_2022_9677_MOESM2_ESM.png]

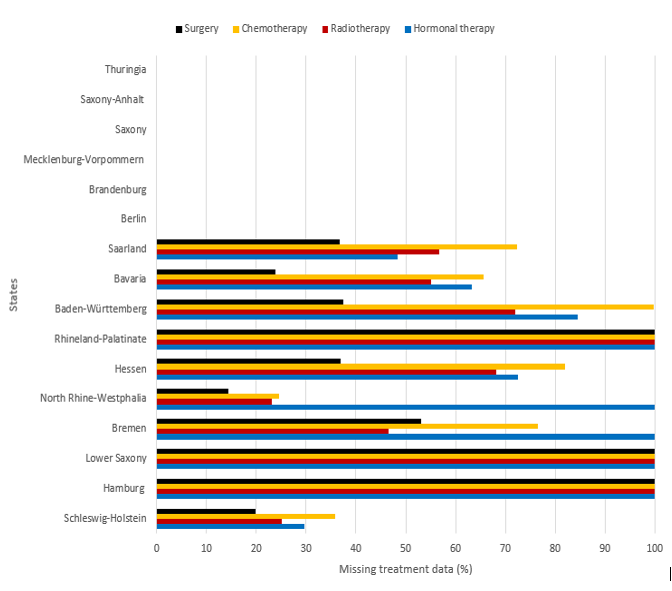

Supplement: Supplementary file 5 — Additional file 5 Proportions of missing treatment data among non-metastatic PCa cases stratified by German federal states, 2005–2015 (n = 263,839) [file 12885_2022_9677_MOESM5_ESM.png]

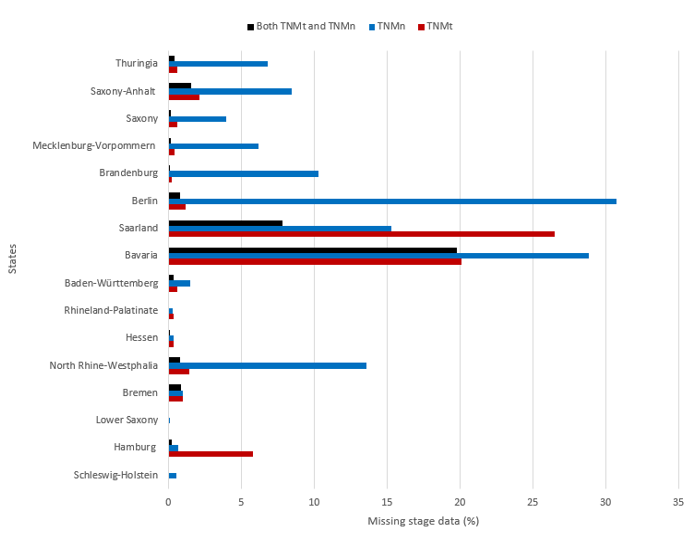

Supplement: Supplementary file 7 — Additional file 7 Proportions of missing TNM stage data in non-metastatic PCa cases stratified by German federal states, 2005–2015 (n = 263, 839) [file 12885_2022_9677_MOESM7_ESM.png]

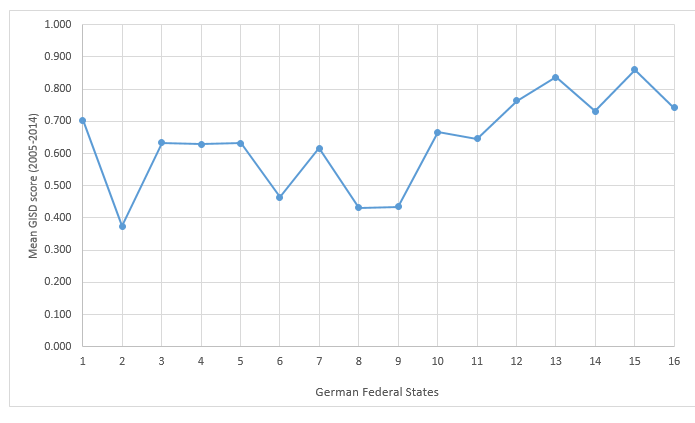

Supplement: Supplementary file 10 — Additional file 10. Mean GISD score of 16 German federal states based on 263,774 PCa cases diagnosed during 2005–2014 (1 = Schleswig-Holstein, 2 = Hamburg, 3 = Lower Saxony, 4 = Bremen, 5 = North Rhine-Westphalia, 6 = Hessen, 7 = Rhineland-Palatinate, 8 = Baden-Württemberg, 9 = Bavaria, 10 = Saarland, 11 = Berlin, 12 = Brandenburg, 13 = Mecklenburg-Vorpommern, 14 = Saxony, 15 = Saxony-Anhalt, and 16 = Thuringia) [file 12885_2022_9677_MOESM10_ESM.png]
