# Supplementary material for: Suboptimal use of hormonal therapy among German men with localized high-risk prostate Cancer during 2005 to 2015: analysis of registry data
Source: BMC Cancer. 2022 Jun 7;22:624. doi: 10.1186/s12885-022-09677-z (PMC9171996; doi:10.1186/s12885-022-09677-z)
Supplement: Supplementary file 3 — Additional file 3 Factors associated with HT use among poorly differentiated and locally advanced PCa cases in seven states which received RT between 2005 and 2014 (n = 2, 648). [file 12885_2022_9677_MOESM3_ESM.docx]

| **Variables** | **Received HT** | | **Use of HT^a^** | |
| --- | --- | --- | --- | --- |
|  | **No**  **(n, %)** | **Yes**  **(n, %)** | **Crude Risk Ratio (95% CI)** | **Adjusted Risk Ratio (95% CI)** |
| **Age (10 year increase)** |  |  | 1.09 (1.02, 1.16) | 1.09 (1.03, 1.16) |
| **Tumor grade^b^** |  |  |  |  |
| Low grade | 64 (33.2) | 129 (66.8) | 1.00 | 1.00 |
| High grade | 1,012 (42.9) | 1,347 (57.1) | 0.85 (0.77, 0.95) | 1.06 (0.95, 1.18) |
| **Stage** |  |  |  |  |
| Poorly differentiated | 850 (45.7) | 1, 009 (54.3) | 1.00 | 1.00 |
| Locally advanced | 280 (35.5) | 509 (64.5) | 1.19 (1.11, 1.27) | 1.27 (1.18, 1.36) |
| **German Index of Socioeconomic-deprivation ^c^** |  |  |  |  |
| Most affluent | 8 (28.6) | 20 (71.4) | 1.00 | 1.00 |
| Medium | 547 (43.3) | 715 (56.7) | 0.79 (0.62, 1.01) | 0.75 (0.59, 0.95) |
| Least affluent | 575 (42.3) | 783 (57.3) | 0.81 (0.64, 1.02) | 0.71 (0.55, 0.90) |
| **Era** |  |  |  |  |
| Pre-guideline era | 390 (40.9) | 564 (59.1) | 1.00 | 1.00 |
| Guideline era | 740 (43.7) | 954 (56.3) | 0.95 (0.89, 1.02) | 0.99 (0.93, 1.06) |
| **Federal states** |  |  |  |  |
| Schleswig-Holstein | 238 (43.2) | 314 (56.9) | 1.00 | 1.00 |
| Berlin | 99 (64.7) | 54 (35.3) | 0.62 (0.49, 0.78) | 0.68 (0.54, 0.84) |
| Brandenburg | 227 (36.7) | 392 (63.3) | 1.11 (1.01, 1.22) | 1.11 (1.01, 1.22) |
| Mecklenburg-Vorpommern | 131 (40.7) | 191 (59.3) | 1.04 (0.93, 1.17) | 1.09 (0.96, 1.24) |
| Saxony | 163 (31.2) | 360 (68.8) | 1.21 (1.10, 1.33) | 1.21 (1.10, 1.33) |
| Saxony-Anhalt | 75 (51.4) | 71 (48.6) | 0.85 (0.71, 1.03) | 0.90 (0.75, 1.10) |
| Thuringia | 197 (59.2) | 136 (40.8) | 0.72 (0.62, 0.83) | 0.72 (0.62, 0.83) |

n= number, %= row percentage, **^a^** Risk ratios from univariate and multivariable log-binomial models, grading information was missed for about 3.63% (96) of the 2,648 cases, **^b^** GISD information available only until 2014, RT= Radiotherapy, HT= Hormonal therapy
