# Supplementary material for: Suboptimal use of hormonal therapy among German men with localized high-risk prostate Cancer during 2005 to 2015: analysis of registry data
Source: BMC Cancer. 2022 Jun 7;22:624. doi: 10.1186/s12885-022-09677-z (PMC9171996; doi:10.1186/s12885-022-09677-z)
Supplement: Supplementary file 4 — Additional file 4 Factors associated with non-treatment among poorly differentiated (n = 3, 243) and locally advanced (n = 1, 690) PCa cases diagnosed between 2005 and 2014. [file 12885_2022_9677_MOESM4_ESM.docx]

| **Variables** | **None-use of both RT and HT in poorly differentiated** | | **None-use of both RT and HT in locally advanced** | |
| --- | --- | --- | --- | --- |
|  | **Crude Risk Ratio (95% CI)** | **Adjusted Risk Ratio (95% CI)** | **Crude Risk Ratio (95% CI)** | **Adjusted Risk Ratio (95% CI)** |
| **Age (10 year increase)** | 0.80 (0.71, 0.90) | 0.79 (0.71, 0.88) | 0.88 (0.74, 1.04) | 0.84 (0.71, 0.99) |
| **German Index of Socioeconomic-deprivation^c^** |  |  |  |  |
| Most affluent | 1.00 | 1.00 | 1.00 | 1.00 |
| Medium | 1.35 (0.53, 3.39) | 0.71 (0.29, 1.73) | 1.37 (0.37, 5.00) | 0.83 (0.24, 2.87) |
| Least affluent | 1.74 (0.69, 4.36) | 0.83 (0.34, 2.03) | 1.23 (0.34, 4.51) | 0.76 (0.22, 2.65) |
| **Era** |  |  |  |  |
| Pre-guideline era | 1.00 | 1.00 | 1.00 | 1.00 |
| Guideline era | 1.35 (1.16, 1.57) | 1.42 (1.22, 1.65) | 1.16 (0.94, 1.44) | 1.15 (0.94, 1.41) |
| **Federal states** |  |  |  |  |
| Brandenburg | 1.00 | 1.00 | 1.00 | 1.00 |
| Schleswig-Holstein | 0.19 (0.11, 0.32) | 0.18 (0.11, 0.31) | 0.11 (0.03, 0.43) | 0.10 (0.03, 0.41) |
| Mecklenburg-Vorpommern | 1.52 (1.22, 1.90) | 1.40 (1.12, 1.76) | 2.23 (1.55, 3.21) | 2.29 (1.57, 3.33) |
| Saxony | 1.50 (1.25, 1.81) | 1.52 (1.26, 1.82) | 2.36 (1.75, 3.17) | 2.31 (1.69, 3.15) |
| Thuringia | 1.41 (1.10, 1.80) | 1.33 (1.04, 1.71) | 1.90 (1.30, 2.77) | 1.81 (1.23, 2.65) |

n= number, %= row percentage, **^a^** grading information was missed for about 2.98% (70) of the 2,349 cases, **^b^** GISD information available only until 2014
