# Supplementary material for: Suboptimal use of hormonal therapy among German men with localized high-risk prostate Cancer during 2005 to 2015: analysis of registry data
Source: BMC Cancer. 2022 Jun 7;22:624. doi: 10.1186/s12885-022-09677-z (PMC9171996; doi:10.1186/s12885-022-09677-z)
Supplement: Supplementary file 6 — Additional file 6 Multivariable binary logistic regression analysis showing predictors of missing hormonal treatment data in Schleswig-Holstein (n = 767) [file 12885_2022_9677_MOESM6_ESM.docx]

| **Variables** | HT information | | Missing HT data |
| --- | --- | --- | --- |
|  | Documented (n, %) | Missing (n, %) | Odds ratio (95%CI) |
| Age at diagnosis (10 year increase) |  |  | 0.40 (0.23, 0.69) |
| Year of diagnosis |  |  |  |
| 2005-2010 | 359 (97.5) | 9 (2.5) | 1.00 |
| 2011-2015 | 379 (95.0) | 20 (5.0) | 2.70 (1.12, 6.51) |
| RT treatment information |  |  |  |
| documented | 726 (97.2) | 21 (2.8) | 1.00 |
| Missing | 12 (60.0) | 6 (40.0) | 26.5 (8.92, 78.67) |
