# Supplementary material for: Suboptimal use of hormonal therapy among German men with localized high-risk prostate Cancer during 2005 to 2015: analysis of registry data
Source: BMC Cancer. 2022 Jun 7;22:624. doi: 10.1186/s12885-022-09677-z (PMC9171996; doi:10.1186/s12885-022-09677-z)
Supplement: Supplementary file 8 — Additional file 8 Multivariable binary logistic regression analyses showing predictors of missing stage data in five states, 2005–2015 (n = 74,098) [file 12885_2022_9677_MOESM8_ESM.docx]

| **Variables** | All five states combined  (n= 74, 098) | Schleswig-Holstein  (n= 9, 546) | Brandenburg (n= 16, 805 ) | Mecklenburg-Vorpommern (n= 8, 979) | Saxony  (n= 27, 755) | Thuringia  (n= 11, 013) |
| --- | --- | --- | --- | --- | --- | --- |
|  | Missing stage information | | | | | |
|  | Odds ratio (95%CI) | Odds ratio (95%CI) | Odds ratio (95%CI) | Odds ratio (95%CI) | Odds ratio (95%CI) | Odds ratio (95%CI) |
| Age at diagnosis (10 year increase) | 3.84  (3.04, 4.86) | 1.54  (0.23, 10.5) | 4.75  (2.16, 10.46) | 2.65  (1.28, 5.45) | 4.50  (3.12, 6.47) | 4.06  (2.71, 6.09) |
| Diagnosis year  2005-2010 | 1.00 | 1.00 | 1.00 | 1.00 | 1.00 | 1.00 |
| Diagnosis year  2011-2015 | 0.25  (0.17, 0.38) | -^*^ | 0.32  (0.09, 1.16) | 0.31  (0.10, 0.97) | 0.24  (0.13, 0.46) | 0.18  (0.08, 0.38) |

^*^Schleswig-Holstein had very few missing stage cases (0.02%), and did not generate estimate for the variable “diagnosis year” because Schleswig-Holstein had no recorded missing stage for the reference-year category: “Missing stage” represents missing values in both T and N stages.
