# Supplementary material for: Suboptimal use of hormonal therapy among German men with localized high-risk prostate Cancer during 2005 to 2015: analysis of registry data
Source: BMC Cancer. 2022 Jun 7;22:624. doi: 10.1186/s12885-022-09677-z (PMC9171996; doi:10.1186/s12885-022-09677-z)
Supplement: Supplementary file 9 — Additional file 9 Multivariable binary logistic regression analyses showing predictors of missing histopathological tumor grade data in five states, 2005–2015 (n = 74,098) [file 12885_2022_9677_MOESM9_ESM.docx]

| **Variables** | All five states combined  (n= 74, 098) | Schleswig-Holstein  (n= 9, 546) | Brandenburg (n= 16, 805 ) | Mecklenburg-Vorpommern (n= 8, 979) | Saxony  (n= 27, 755) | Thuringia  (n= 11, 013) |
| --- | --- | --- | --- | --- | --- | --- |
|  | Missing histopathological tumor grade information | | | | | |
|  | Odds ratio (95%CI) | Odds ratio (95%CI) | Odds ratio (95%CI) | Odds ratio (95%CI) | Odds ratio (95%CI) | Odds ratio (95%CI) |
| Age at diagnosis (10 year increase) | 1.14  (1.11, 1.17) | 1.27  (1.14, 1.41) | 1.07  (1.00, 1.14)) | 0.99  (0.92, 1.06) | 1.07  (1.03, 1.11) | 1.26  (1.15, 1.39) |
| Diagnosis year  2005-2010 | 1.00 | 1.00 | 1.00 | 1.00 | 1.00 | 1.00 |
| Diagnosis year  2011-2015 | 1.52  (1.47, 1.59) | 2.35  ( 2.00, 2.76) | 3.38  (3.03, 3.76) | 1.71  (1.53, 1.90) | 1.22  (1.15, 1.29) | 0.83  (0.72, 0.95) |
