# Supplementary material for: Suboptimal use of hormonal therapy among German men with localized high-risk prostate Cancer during 2005 to 2015: analysis of registry data
Source: BMC Cancer. 2022 Jun 7;22:624. doi: 10.1186/s12885-022-09677-z (PMC9171996; doi:10.1186/s12885-022-09677-z)
Supplement: Supplementary file 11 — Additional file 11. Treatment recommendations of German S3-Guideline and EAU guidelines for localized high-risk prostate cancer patients, 2005 to 2021 [file 12885_2022_9677_MOESM11_ESM.docx]

Additional file 11: Treatment recommendations of German S3-Guideline and EAU guidelines for localized high-risk prostate cancer patients, 2005 to 2021

| German S3 and EAU-guidelines along with their different versions | Definition of  High-risk Prostate cancer | | Treatment recommendations for combinations of hormonal- and radiotherapy | |
| --- | --- | --- | --- | --- |
|  | Localized High-risk | Locally advanced | Localized High-risk | Locally advanced |
| German S3-Guideline for Prostate Cancer: Version 1. (September 2009)(1) | PSA> 20 ng/ml, or biopsy GS ≥8, or clinical stage of ≥ T2c | T_3-4_N_0_M_0_ | Radiotherapy (RT) plus 2-3 months neoadjuvant and/or adjuvant hormonal therapy (HT) of at least 2 years, preferably 3 years (#pp65) | RT plus 2-3 months neoadjuvant and/or adjuvant HT of at least 2 years, preferably 3 years (#pp82-83) |
| German S3-Guideline for Prostate Cancer: Version 1.01 (October 2009)(2) | PSA> 20 ng/ml, or biopsy GS ≥8, or clinical stage of ≥ T2c | T_3-4_N_0_M_0_ | RT plus 2-3 months neoadjuvant and/or adjuvant HT of at least 2 years, preferably 3 years (#pp69) | RT plus 2-3 months neoadjuvant and/or adjuvant HT of at least 2 years, preferably 3 years (#pp86-87) |
| German S3-Guideline for Prostate Cancer: Version 1.03 (March 2011)(3) | PSA> 20 ng/ml, or biopsy GS ≥8, or clinical stage of ≥ T2c | T_3-4_N_0_M_0_ | RT plus 2-3 months neoadjuvant and/or adjuvant HT of at least 2 years, preferably 3 years (#pp69) | RT plus 2-3 months neoadjuvant and/or adjuvant HT of at least 2 years, preferably 3 years (#pp86-87)  -HDR-brachytherapy combined with percutaneous RT is an option (#pp 94) |
| German S3-Guideline for Prostate Cancer: Version 2.0 (May 2012)(4) | PSA> 20 ng/ml, or biopsy GS ≥8, or clinical stage of ≥ T2c | T_3-4_N_0_M_0_ | RT plus 6 months neoadjuvant and/or adjuvant hormonal therapy of 2- 3 years (#pp95)  - Duration of HT depends on comorbidity, age, life expectancy and tumor extent (#pp95)  - HDR-Brachytherapy combined with percutaneous RT is an option, and the value of additional HT was not clarified (#pp105, 145-144) | RT plus 2-3 months neoadjuvant and/or adjuvant HT of at least 2 years, preferably 3 years (#pp120-87)  -HDR-brachytherapy combined with percutaneous RT is an option for cT3 (#pp 125) |
| German S3-Guideline for Prostate Cancer: Version 3.0 (September 2014) and 3.01 (October 2014)(5) | PSA> 20 ng/ml, or biopsy GS ≥8, or clinical stage of ≥ T2c | T_3-4_N_0_M_0_ | RT plus up to 6 months of neoadjuvant and/or adjuvant hormonal therapy of 2- 3 years (#pp100-102)  - Duration of HT depends on comorbidity, age, life expectancy and tumor extent (#pp95)  - HDR-Brachytherapy combined with percutaneous RT is an option, and the value of additional HT was not clarified (#pp105, 111-112) | RT plus up to 6 months of neoadjuvant and/or adjuvant HT of at least 2 years, preferably 3 years (#pp126-127)  - HDR-brachytherapy combined with percutaneous RT is an option for cT3 (#pp 132) |
| German S3-Guideline for Prostate Cancer: Version 4.0 (December 2016)(6) | PSA> 20 ng/ml, or biopsy GS ≥8, or clinical stage of ≥ T2c | T_3-4_N_0_M_0_ | RT plus 6 months neoadjuvant and/or adjuvant hormonal therapy of 2- 3 years (#pp106-108)  - Duration of HT depends on comorbidity, age, life expectancy and tumor extent (#pp95)  - HDR-Brachytherapy combined with percutaneous RT is an option, and the value of additional HT was not clarified (#pp105, 108) | RT plus up to 6 months of neoadjuvant and/or adjuvant HT of at least 2 years, preferably 3 years (#pp133-134)  - HDR-brachytherapy combined with percutaneous RT is an option for cT3 (#pp 139) |
| German S3-Guideline for Prostate Cancer: Version 5.0 (April 2018) and 5.1 (May 2019)(7) | PSA> 20 ng/ml, or biopsy GS ≥8, or clinical stage of ≥ T2c | T_3-4_N_0_M_0_ | RT plus 6 months neoadjuvant and/or adjuvant hormonal therapy of 2- 3 years (#pp138-140)  - HDR-Brachytherapy combined with percutaneous RT is an option, and the value of additional HT was not clarified (#pp123) | RT plus up to 6 months of neoadjuvant and/or adjuvant HT of at least 2 years, preferably 3 years (#pp138-139)  - HDR-brachytherapy combined with percutaneous RT is an option for cT3 (#pp 143) |
| German S3-Guideline for Prostate Cancer: Version 6.0 (May 2021)(8) | PSA> 20 ng/ml, or biopsy GS ≥8, or clinical stage of ≥ T2c | T_3-4_N_0_M_0_ | RT plus 6 months neoadjuvant and/or adjuvant hormonal therapy of 2- 3 years (#pp108)  - HDR-Brachytherapy combined with percutaneous RT is an option, and the value of additional HT was not clarified (#pp123) | RT plus up to 6 months of neoadjuvant and/or adjuvant HT of at least 2 years, preferably 3 years (#pp144-145)  - HDR-brachytherapy combined with RT is an option for cT3 (#pp 148) |
| EAU-guideline on Prostate Cancer versions 2005 - 2011 (9) ^,^(10)^,^(11)^,^(12)^,^ (13)^,^ (14) | PSA> 20 ng/ml, or biopsy GS ≥8, or clinical stage of ≥ T2c (≥T3a for cases eligible to prostatectomy) | T_3-4_N_0-x_M_0_ , or  T_1-4_N_+_M_0_ | Short-term HT before and during RT | RT in combination with concomitant and adjuvant HT of 2 to 3 years |
| EAU-guideline on Prostate Cancer versions 2012-2017 (15)^,^(16)^,^ (17)^,^ (18)^,^ (19)^,^ (20) | PSA> 20 ng/ml, or biopsy GS ≥8, or clinical stage of ≥ T2c-3ca (≥T3a for cases eligible to prostatectomy), or CAPRA 6-10 , or ISUP Grade_4-5_ | T_3-4_N_0-x_M_0_ , or  T_3b-4_N_0_M_0 ,_ or  T_1-4_N_+_M_0_ | Long-term HT before and during RT  - Duration of HT depends on comorbidities, and the number of poor prognostic factors | RT in combination with concomitant and adjuvant HT of 2-3 years with a WHO 0-2 performance status |
| EAU-ESTRO-ESUR-SIOG guideline on prostate cancer on Prostate Cancer versions 2018-2020 (21)^,^ (22)^,^ (23)^,^ | PSA> 20 ng/ml, or biopsy GS ≥8, or clinical stage of ≥ T2c (≥T3a for cases eligible to prostatectomy) | T_3-4_N_0-x_M_0_ , or  T_1-4_N_+_M_0_ | Long-term HT before and during RT  - Duration of HT depends on comorbidities, and the number of poor prognostic factors | RT in combination with concomitant and adjuvant HT of 2-3 years with a WHO 0-2 performance status |
| Peer reviewed publications underlying EAU-guideline on Prostate Cancer: versions 2005 & 2008  (Based on literature review of 2004 to 2007) (24)^,^ (25) |  |  | HT (2 - 3 years) for poorly differentiated cases + RT | - HT (2 - 3 years) + RT  - HT + RT was not a standard option for N_+_M_0_  in the 2005 and 2008 publications |
| Peer reviewed publications underlying EAU-guideline on Prostate Cancer: version 2011(Based on literature review of 2007 to 2010) (26) |  |  | Short-term HT before and during RT improved overall survival, but 3 years adjuvant HT was recommended | Concomitant and adjuvant HT (3 years) + RT |
| Peer reviewed publications underlying EAU-guideline on Prostate Cancer: versions 2014  (Based on literature review of 2011 to 2013) (27) |  |  | Long-term HT before and during RT | Concomitant and adjuvant HT (3 years) + RT for patients with WHO 0-2 performance status |

EAU= European Association of Urology, CAPRA= Cancer of the Prostate Risk Assessment; ISUP= = International Society for Urological Pathology; GS= Gleason Score; PSA= Prostate-specific antigen

# References

1. Guideline program oncology (German Cancer Society GCA, AWMF). “Interdisciplinary guideline of the quality S3 for the early detection, diagnosis and therapy of the different stages of prostate carcinoma “ [Internet]. 2009. Available from: https://www.leitlinienprogramm-onkologie.de/leitlinien/prostatakarzinom/
2. Guideline program oncology (German Cancer Society GCA, AWMF). “Interdisciplinary guideline of the quality S3 for the early detection, diagnosis and therapy of the different stages of prostate carcinoma: Version 1.01“ [Internet]. 2009. Available from: https://www.leitlinienprogramm-onkologie.de/fileadmin/user_upload/Downloads/Leitlinien/Prostatatkarzinom/S3LLPCa_091002.pdf
3. Guideline program oncology (German Cancer Society GCA, AWMF). “Interdisciplinary guideline of the quality S3 for the early detection, diagnosis and therapy of the different stages of prostate carcinoma: Version 1.03” [Internet]. 2011. Available from: https://www.leitlinienprogramm-onkologie.de/fileadmin/user_upload/Downloads/Leitlinien/Prostatatkarzinom/S3_LL_PCa_-110331_Version1.03.pdf
4. Guideline program oncology (German Cancer Society GCA, AWMF). “Interdisciplinary guideline of the quality S3 for the early detection, diagnosis and therapy of the different stages of prostate carcinoma: Version 2.0” [Internet]. 2012. Available from: https://www.leitlinienprogramm-onkologie.de/fileadmin/user_upload/Downloads/Leitlinien/Prostatatkarzinom/S3-Prostatakarzinom-OL-Langversion.pdf
5. Guideline program oncology (German Cancer Society GCA, AWMF). “Interdisciplinary guideline of the quality S3 for the early detection, diagnosis and therapy of the different stages of prostate carcinoma: Versions 3.0 and 3.01” [Internet]. 2014. Available from: https://www.leitlinienprogramm-onkologie.de/fileadmin/user_upload/Downloads/Leitlinien/Prostata_3.0/LL_Prostata_Langversion_3.0.pdf
6. Guideline program oncology (German Cancer Society GCA, AWMF). “Interdisciplinary guideline of the quality S3 for the early detection, diagnosis and therapy of the different stages of prostate carcinoma: Versions 4.0” [Internet]. 2016. Available from: https://www.leitlinienprogramm-onkologie.de/fileadmin/user_upload/Downloads/Leitlinien/Prostata_4_0/LL_Prostata_Langversion_4.0.pdf
7. Guideline program oncology (German Cancer Society GCA, AWMF). “Interdisciplinary guideline of the quality S3 for the early detection, diagnosis and therapy of the different stages of prostate carcinoma: Versions 5.0 and 5.1.” 2018.
8. Guideline program oncology (German Cancer Society GCA, AWMF). S3 Guideline Prostate Cancer [Internet]. 2021. Available from: https://www.leitlinienprogramm-onkologie.de/fileadmin/user_upload/Downloads/Leitlinien/Prostatatkarzinom/Version_6/LL_Prostatakarzinom_Langversion_6.0.pdf
9. Aus G, Abbou CC, Bolla M, Heidenreich A, van Poppel H, Schmid H-P, Wolff JM, et al. EAU guidelines on prostate cancer [Internet]. 2005. Available from: <https://uroweb.org/wp-content/uploads/EAU-Guidelines-on-Prostate-Cancer-2005.pdf>
10. Heidenreich A, Aus G, Abbou CC, Bolla M, Joniau S, Matveev V, et al. EAU guidelines on prostate cancer [Internet]. 2007. Available from: <https://uroweb.org/wp-content/uploads/07-Prostate-Cancer-6.pdf>
11. Heidenreich A, Aus G, Abbou CC, Bolla M, Joniau S, Matveev V, et al. EAU guideline on prostate cancer [Internet]. 2008. Available from: <https://uroweb.org/wp-content/uploads/07-Prostate-Cancer-3.pdf>
12. Heidenreich A, Bolla M, Joniau S, van der Kwast TH, Matveev V, Mason MD, et al. EAU guideline on prostate cancer [Internet]. 2009. Available from: https://uroweb.org/wp-content/uploads/05-Prostate-Cancer.pdf
13. Heidenreich A, Bolla M, Joniau S, Mason MD, Matveev V, Mottet N, at al. EAU guideline on prostate cancer [Internet]. 2010. Available from: https://uroweb.org/wp-content/uploads/EAU-Guidelines-Prostate-Cancer-2010.pdf
14. Heidenreich A, Bolla M, Joniau S, Mason MD, Matveev V, Mottet N, et al. Guideline on Prostate Cancer. Update [Internet]. 2011; Available from: https://uroweb.org/wp-content/uploads/08_Prostate_Cancer-September-22nd-2011.pdf
15. Heidenreich A, Bastian PJ, Bellmunt J, Bolla M, Joniau S, Mason MD, et al. EAU guideline on prostate cancer [Internet]. 2012. Available from: https://uroweb.org/wp-content/uploads/08-Prostate-Cancer_LR-March-13th-20122.pdf
16. Heidenreich A, Bastian PJ, Bellmunt J, Bolla M, Joniau S, Mason MD, et al. Wiegel FZ. EAU guideline on prostate cancer [Internet]. 2013. Available from: https://uroweb.org/wp-content/uploads/09_Prostate_Cancer_LR.pdf
17. Mottet N, Bastian PJ, Bellmunt J, van den Bergh RCN, Bolla M, van Casteren NJ, et al. EAU guideline on prostate cancer [Internet]. 2014. Available from: https://uroweb.org/wp-content/uploads/1607-Prostate-Cancer_LRV3.pdf
18. Mottet N, Bellmunt J, Briers E, van den Bergh RCN, Bolla M, van Casteren NJ, et al. EAU guideline on prostate cancer [Internet]. 2015. Available from: https://uroweb.org/wp-content/uploads/EAU-Guidelines-Prostate-Cancer-2015-v2.pdf
19. Mottet N, Bellmunt J, Briers E, Bolla M, Cornford P, De Santis PM, et al. EAU-ESTRO-ESUR-SIOG guideline on prostate cancer [Internet]. 2016. Available from: https://uroweb.org/wp-content/uploads/EAU-Guidelines-Prostate-Cancer-2016-1.pdf
20. Mottet N, Bellmunt J, Briers E, Bolla M, Bourke L, Cornford P, et al. Cumberbatch PMW. EAU-ESTRO-ESUR-SIOG guideline on prostate cancer [Internet]. 2017. Available from: https://uroweb.org/wp-content/uploads/09-Prostate-Cancer_2017_web.pdf
21. Mottet N, van den Bergh RCN, Briers E, Bourke L, Cornford P, De Santis M, et al. EAU-ESTRO-ESUR-SIOG guideline on prostate cancer [Internet]. 2018. Available from: https://uroweb.org/wp-content/uploads/EAU-ESUR-ESTRO-SIOG-Guidelines-on-Prostate-Cancer-large-text-V2.pdf
22. Mottet N, van den Bergh RCN, Briers E, Cornford P, De Santis M, Fanti S, et al. EAU-ESTRO-ESUR-SIOG guideline on prostate cancer [Internet]. 2019. Available from: https://uroweb.org/wp-content/uploads/EAU-EANM-ESUR-ESTRO-SIOG-Guidelines-on-Prostate-Cancer-2019-1.pdf
23. Mottet N, Cornford P, van den Bergh RCN, Briers E, De Santis M, Fanti S, et al. EAU-ESTRO-ESUR-SIOG guideline on prostate cancer [Internet]. 2020. Available from: https://uroweb.org/wp-content/uploads/EAU-EANM-ESTRO-ESUR-SIOG-Pocket-Guidelines-on-Prostate-Cancer-2020.pdf
24. Aus G, Abbou CC, Bolla M, Heidenreich A, Schmid HP, Van Poppel H, et al. EAU guideline on prostate cancer. Eur Urol. 2005; 48(4): 546-51.
25. Heidenreich A, Aus G, Bolla M, Joniau S, Matveev VB, Schmid HP, et al. EAU Guidelines on Prostate Cancer. Eur Urol. 2008; 53(1): 68-80.
26. Heidenreich A, Bellmunt J, Bolla M, Joniau S, Mason M, Matveev V, et al. EAU guidelines on prostate cancer. Part 1: Screening, diagnosis, and treatment of clinically localised disease. Eur Urol. 2011; 59(1):61-71.
27. Heidenreich A, Bastian PJ, Bellmunt J, Bolla M, Joniau S, Van Der Kwast T, et al. EAU guidelines on prostate cancer. Part 1: Screening, diagnosis, and local treatment with curative intent - Update 2013. Eur Urol. 2014; 65(1): 124-37.
